# Dental extraction in patients receiving dual antiplatelet therapy

**DOI:** 10.4317/medoral.20510

**Published:** 2015-08-04

**Authors:** Paulino Sánchez-Palomino, Paulino Sánchez-Cobo, Alberto Rodriguez-Archilla, Maximino González-Jaranay, Gerardo Moreu, José-Luis Calvo-Guirado, Miguel Peñarrocha-Diago, Gerardo Gómez-Moreno

**Affiliations:** 1 Dental graduate, Master’s Program in Periodontology and Implant Dentistry, Faculty of Dentistry, University of Granada, Granada, Spain; 2 Doctor of Dentistry, Head of Dental Medicine Service, Hospital Princesa de Jaén, Jaén, Spain; 3 Department of Buccal Medicine, Faculty of Dentistry, University of Granada, Granada, Spain; 4 Department of Periodontics, Master’s Program in Periodontology and Implant Dentistry, Faculty of Dentistry, University of Granada, Granada, Spain; 5 Full Professor in Dentistry, Director of International Research Cathedra in Dentistry, San Antonio Catholic University of Murcia, UCAM, Murcia, Spain; 6 Chairman of Oral Surgery, Department of Stomatology, Valencia University Medical and Dental School, Spain; 7 Department of Special Care in Dentistry, Master’s Program in Periodontology, Implant Dentistry, and Pharmacological Research, Faculty of Dentistry, University of Granada, Granada, Spain

## Abstract

**Background:**

Dual anti platelet therapy consists of administering antiplatelet (antiaggregant) drugs (clopidogrel and aspirin) to prevent thrombotic processes, as a preventative measure in patients with acute coronary disease, or in patients subjected to percutaneous coronary intervention.

**Objectives:**

The purpose of this study was to evaluate the efficacy of a protocol for performing dental extraction in patients receiving dual anti platelet therapy.

**Material and Methods:**

Thirty-two patients undergoing dental extractions were included in the study. The variables evaluated were: collagen-epinephrine fraction, collagen- adenosine diphosphate fraction, surgical surface, post-surgical measures, and adverse effects. Alveolar sutures and gauzes impregnated with an antifibrinolytic agent (tranexamic acid), which the patient pressed in place for 30 minutes, were applied to all patients as post-surgical measures. Descriptive statistics were calculated and analyzed with Student’s t-test to compare pairs of quantitative variables; simple regression analysis was performed using Pearson’s correlation coefficient. Statistical significance was set at *p*<0.05.

**Results:**

Collagen/epinephrine fraction was 264.53±55.624 seconds with a range of 135 to 300 seconds, and collagen/ADP fraction was 119.41±44.216 seconds, both values being higher than normal. As a result of the post-surgical measures taken, no patients presented postoperative bleeding, hematoma or infection.

**Conclusions:**

Dental extraction was safe for patients receiving dual anti-platelet therapy when using sutures and gauze impregnated with tranexamic acid, which the patient pressed in place for 30 minutes.

** Key words:** Aspirin, clopidogrel, tranexamic acid, dental extraction, platelet function.

## Introduction

Hemostasis is the set of mechanisms that impede the loss of blood through fibrin formation (clotting). This process consists of three phases: i) vascular phase, in which transitory neurogenic vasoconstriction is produced, reducing the escape of blood (for a duration of about 20 seconds); ii) platelet phase, in which platelet thrombus formation takes place at the same time as platelet aggregation, which concentrates a large number of factors necessary for the third phase; iii) plasma coagulation consists of the complex sequence of proteolytic reactions that bring about fibrin clotting, with clots beginning to develop within 15-20 seconds. This process is initiated by activating substances secreted by blood vessel, platelets and blood proteins adhering to blood vessel walls, known as coagulation factors (1).

Dual anti platelet therapy consists of the combination of two antiaggregant drugs (usually clopidogrel and aspirin) and has two indications: the prevention of thrombotic processes (cerebrovascular accidents, CVA), and the prevention of acute myocardial infarction (AMI) in patients with acute coronary syndromes or patients subjected to percutaneous coronary intervention (stent) (2,3).

Platelets play a central role in the pathogeny of thrombotic processes; platelet inhibiting drugs are used to prevent these processes. Widespread research has shown that aspirin produces undisputed benefits in the secondary prevention of vascular complications. Aspirin’s anti platelet action is due mainly to the irreversible inhibition of cyclooxigenase activity by acetylation of this enzyme’s serine hydroxyl group (4).

The search for other drugs with few adverse effects has led to research into clopidogrel, an anti platelet agent derived from thienopyridine that antagonizes platelet aggregation induced by adenosine diphosphate (ADP). Clopidogrel was evaluated in a 1996 CAPRIE (clopidogrel versus aspirin in patients at risk of ischaemic events) trial (5) that compared a 325 mg daily dose of aspirin with 75 mg of clopidogrel daily. Clopidogrel was found to be superior to aspirin in the prevention of the combined risk of CVA, AMI, and cardiovascular mortality. But when individual complications were analyzed separately, it was found that this superiority was only maintained in the patient group with symptomatic peripheral arterial disease (PAD). As aspirin and clopidogrel have different mechanisms of action, it was thought that the combination of the two would boost the prevention of cardiovascular complications (6). When patients receiving antiplatelet therapies require dental extractions, it is essential to ensure adequate hemorrhage and hematoma management. Oral surgery procedures can be modified to minimize the risk of intra- and postoperative bleeding.

In this context, the objective of this study was to evaluate the efficacy of a protocol for performing dental extraction in patients receiving dual anti platelet therapy.

## Material and Methods

-Patients 

The study included 32 patients attending the Dental Medicine Service of Jaén Hospital’s Neurotrauma Center (Jaén, Spain) who were to undergo dental extractions. The subjects were all patients who had suffered myocardial infarction or heart disease necessitating coronary revascularization and stent placement within the preceding year. All patients had been prescribed two platelet antiaggregants (clopidogrel and aspirin) as prevention against thromboembolic accidents. All subjects gave their informed consent to take part and their full medical histories were recorded. Patients prescribed any medication that would alter hemostasis were excluded from the trial, as were those with terminal diseases, or with major risks of mortality, or who needed extractions involving osteotomies. The protocol of the study was in accordance with the Declaration of Helsinki and was approved by the Ethics Committee of University of Granada (nº FOD/GR/29/2011).

-Variables

Variables analyzed in the trial were: collagen-epinephrine fraction, collagen-ADP fraction, surgical surface, post-surgical measures, and adverse effects.

Collagen-epinephrine fraction and collagen-ADP fraction are two figures derived from a test that evaluates platelet function using a platelet function analyzer (PFA-100®, Siemens, Munich, Baveria, Germany) that aspirates blood through two discs containing different agonists to platelets (collagen-adrenalin and collagen-ADP). It measures the time taken to close the discs internal orifice (obturation time). Normal values range between 71 and 119 seconds for the disc with collagen/ADP. The platelet function test times are prolonged in cases of thrombocytopenia or thrombopathia, and so the delay in obturation with collagen/adrenalin is characteristic of patients who consume aspirin or other non-steroidal anti-inflammatory drugs (NSAIDs), while the prolongation of obturation time with collagen-ADP occurs in patients consuming antiaggregant inhibitors of ADP (clopidogrel, dipiridamole) (for example, in patients with von Willebrand disease).

To perform surgical surface evaluation, points were scored for each type of tooth or the alveolar surface it occupied (1 point for upper and lower incisors, 1.5 points for canines and premolars, 2 points for molars) in order to assess a possible relation between the incidence of complications and alveolar surface occupied by the tooth. The same post-surgical measures were applied to all patients. Surgery was not begun until a favorable report (coagulation test results) had been submitted by the hematologist. The local anesthetic used was articaine with 0.5 mg of epinephrine (Ultracain® 0.5%. Normon S.L., Barcelona, Spain), except in patients with intolerance to local anesthetics or excessive response to epinephrine (in which case mepivacaine without epinephrine was used). All alveolae were sutured with 2-0 silk and a curved triangular needle (Normon S.L., Barcelona, Spain), which aimed to close the gingival margins and so improve hemostasis. When sutured, the patient was given gauze impregnated with tranexamic acid (Amchafibrin®, Rottapharm Madaus S.L., Barcelona, Spain), which he/she was asked to bite on for 30 minutes. Afterwards the patient was examined again to ensure that the hemorrhage had been stopped. When this had been established, the patient was given a packet of sterile gauzes and an ampoule of tranexamic acid and sent home. The secondary effects evaluated were: none (0 points); bleeding [1]; hematoma [2]; infection [3]; others [4].

-Statistical analysis 

Descriptive statistics were calculated (arithmetic mean, standard deviation, ranges and percentages). Student’s t-test was applied to compare quantitative variables (pairs of mean values). Simple regression analysis was also applied using the Pearson correlation coefficient. Statistical significance was set at *p*<0.05.

## Results

Of the 32 patients, 13 were men (40.6%) and 19 women (59.4%), with ages ranging from 43 to 84 years (mean age 71.03±9.14 years). The mean value for collagen/epinephrine fraction was 264.53±55.624 seconds with a range of 135-300 seconds, without significant differences in relation to sex (*p*=0.56). The mean value for collagen/ADP fraction was 119.41±44.216 seconds with a range of 95-300 seconds without significant differences in relation to sex (*p*=0.33). Both fraction values were higher than the normal laboratory figures. No patient presented bleeding after surgery. The mean value obtained for surgical surface was 2.016±0.6896 points ranging between 1 and 4 points, without significant differences in relation to sex (*p*=0.68).

The following parameters were found to be unaffected by patient age: collagen/epinephrine fraction (r=0.194; *p*=0.28), collagen/ADP fraction (r=-0.046; p=0.80), surgical surface (r=-0.005; *p*=0.97) ( Table 1). Collagen/epinephrine fraction did not influence either collagen/ADP fraction (r=0.292; *p*=010), or surgical surface (r=-0.028; *p*=0.88) ( Table 2). As a result of the post-surgical measures taken, no patient presented either hematoma or infection after dental extraction.

**Table 1:**
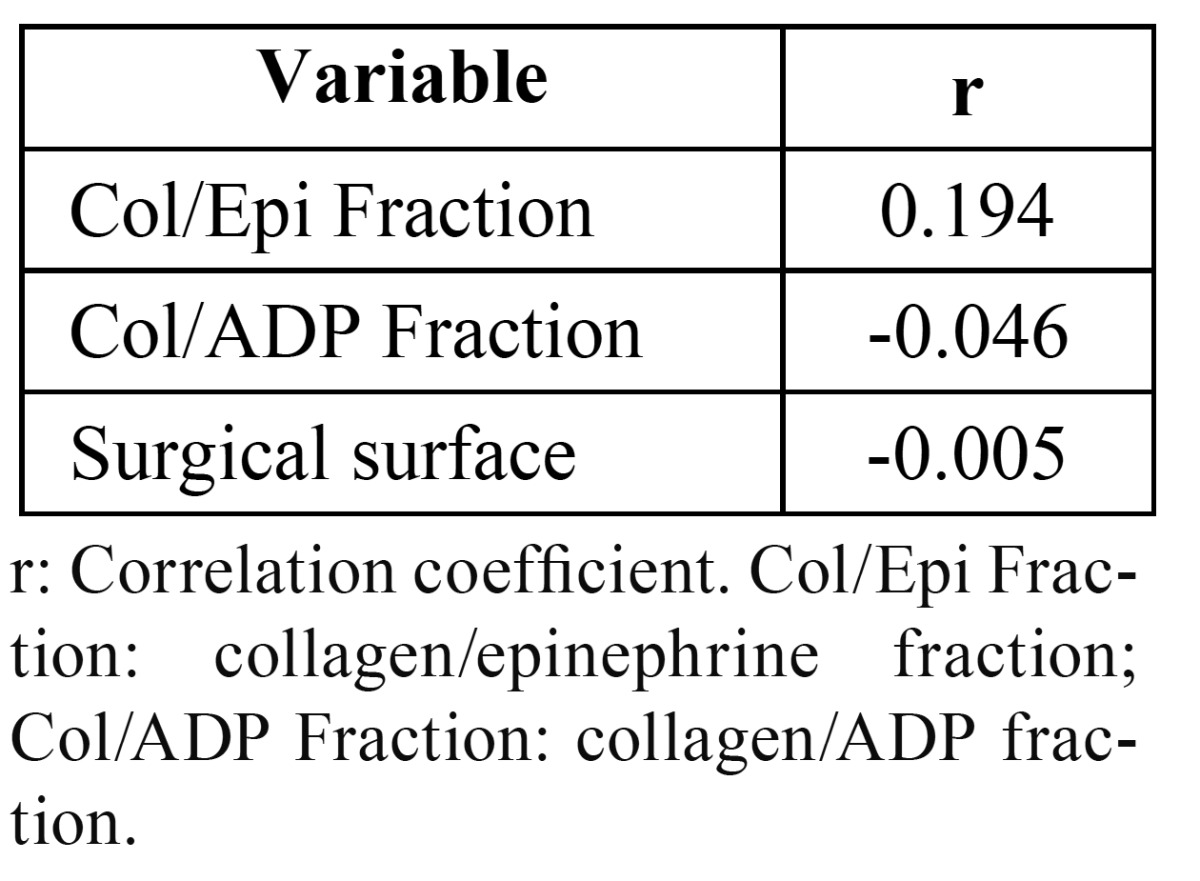
Correlation between age and other variables.

**Table 2:**
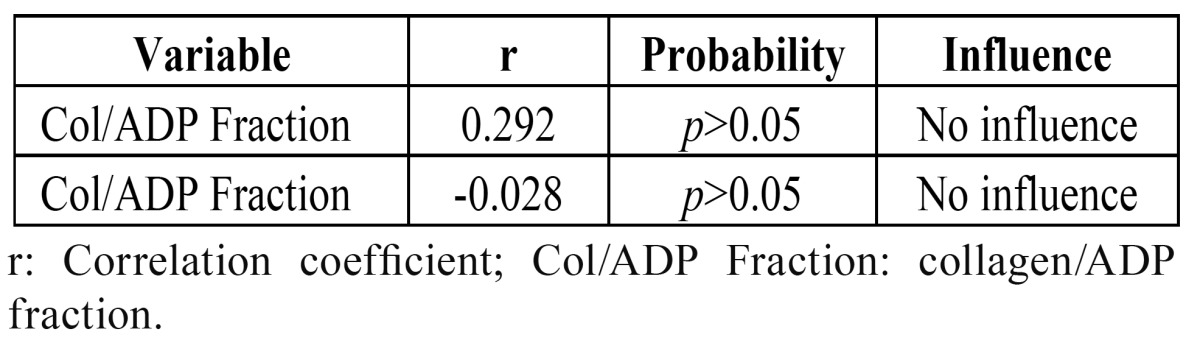
Correlation between Col/Epi fraction and other variables.

## Discussion

Treatment involving oral surgery in patients with platelet function disorders must attend to the nature and severity of the disorder as well as type, localization and extent of the proposed surgical intervention. The level of risk will depend on sufficient access to the surgical site to allow adequate local hemostasis management. Without adequate management, hemorrhage and hematoma can cause obstruction of the airway, placing the patient’s life in danger.

Among the various measures available for minimizing the risk of intra- and postoperative bleeding, the most important are: to minimize trauma; avoid flaps; to use surgical techniques that facilitate suturing; cauterization; all the measures necessary for good hemostasis management; and the removal of all granulation tissue from areas of chronic inflammation (7).

A hematological study (coagulation test) is made prior to oral surgery and this should take place within 24 hours before surgery, although in the case of extractions in series, the test results should remain valid for up to two weeks, taking into consideration the patient’s behavior following each surgical session. Once the two-week period is up, a new coagulation test must be made if further extractions are to be performed.

In the present study of platelet function, collagen/epinephrine and collagen/ADP fractions increased due to the fact that in all cases patients had been prescribed medication that altered platelet function (clopidogrel and aspirin). In 2006, Arrieta-Blanco *et al*. (8) published a study that evaluated the efficacy of the PFA-100 platelet function analyzer. Samples were taken from 33 patients aged between 24 and 80 years, all receiving anti platelet treatment, who underwent oral surgery. They were subjected to a bleeding time test using the Ivy method, an international normalized ratio (INR) test carried out on the same day, and a Coagucheck an hour before surgery, as well as evaluation of bleeding time by the PFA-100. The authors found the PFA-100 platelet function analyzer for the measurement of bleeding time to be more precise in comparison with the traditional Ivy method.

Platelet antiaggregant drugs are associated with increased bleeding time and higher risk of post-operative hemorrhage (9,10). For this reason, some dentists still recommend the interruption of therapy by these drugs (mainly aspirin) at least three days before any oral surgery procedure. But interrupting the use of the drugs exposes the patient to a risk of vascular problems with a potential for severe morbidity (11).

The use of antifibrinolytic agents (tranexamic acid and epsilon aminocaproic acid), is an approach described by many researchers as an element of oral surgery protocols, but there is great disparity in the ways, means and times of their use (12-14). Some authors have recommended the combination of local antifibrinolytic therapy (tranexamic acid) and hemostatic agents as an effective prevention against postoperative hemorrhage (15). Others suggest that many patients can undergo surgical treatments safely without interrupting their usual therapeutic anti-coagulation regime and without any additional medical intervention (16) by using tranexamic acid locally as a postoperative antifibrinolytic agent for two days (17). Meanwhile, others (18) have used human fibrin as a hemostatic agent.

Rakocz *et al*. (19) used a fibrin gel to prevent hemorrhage in patients with hemorrhagic disorders, but the high economic cost restricts this approach. Other research has rejected the use of fibrin gel due to the risk of viral infections (20). Platelets are a natural deposit for growth factors such as platelet-derived growth factor, transformation growth factor beta, insulin-like growth factor, and epithelial growth factor (21). For this reason, many doctors use autologous platelet concentrate to allow the healing process in patients receiving anticoagulation therapy before they undergo cardiovascular surgery, as this runs a high risk of hemorrhage (14).

The present work used a tamponade of gauze soaked in tranexamic acid for 30 minutes; patients were advised to swallow saliva, not to spit, and not to talk during this period. The technique stopped bleeding successfully by retaining blood clots and allowing them to coagulate as well as possible. Rojas et al. simplified the process applying the tamponade of tranexamic acid soaked gauze for ten minutes after extraction (22). Sindet-Pedersen *et al*. also recommend applying gauze soaked in tranexamic acid immediately following dental extraction, applying pressure for several minutes, followed by mouthwashes (4.8% aqueous solution of tranexamic acid) every six hours for seven days (23).

Another controversial issue is the use of surgical sutures, which varies from protocol to protocol. For some researchers, suturing is to be avoided (24,25), and if necessary, nonresorbable sutures should be used, as these prevent inflammatory responses that would have a fibrinolytic action on blood clotting (26). For others, such as Brewer, both resorbable and nonresorbable sutures can be used, depending on the dentist’s individual criteria and skill; the only problems with nonresorbable sutures is that they require a postoperative visit (4-7 days later) and there is a possibility of hemorrhage when the sutures are removed (27). In the present study, suturing was taken as a norm following all extractions. The suture material used was 2-0 silk with a curved triangular needle for better closure of the gingival margins, which was seen to improve hemostasis considerably. The stitches were removed 8-10 days after extraction. The duration of the therapy is another controversial topic, varying in the literature from six months up to two years, twelve months being the most commonly used duration (28).

Lastly, comparing the present results with Napeñas *et al*. (29) and Lillis *et al*. (30), which compared complications arising in patients with simple antiagreggation versus patients receiving dual therapy, the incidence of complications in the present study was very low.

## Conclusion

Dental extraction in patients receiving dual antiagreggant therapy was made safe by using a protocol that kept trauma to a minimum, used non re sorbable sutures, and applied an antifibrinolytic agent (gauze impregnated with tranexamic acid) that the patient held in place under pressure for 30 minutes.
